# Assessing mandibular body changes in growing subjects: a comparison of CBCT and reconstructed lateral cephalogram measurements

**DOI:** 10.1038/s41598-020-68562-6

**Published:** 2020-07-16

**Authors:** Cinzia Maspero, Marco Farronato, Francesca Bellincioni, Davide Cavagnetto, Andrea Abate

**Affiliations:** 1 Department of Biomedical Surgical and Dental Sciences, University of Milan, 20142 Milan, Italy; 2 Fondazione IRCCS Cà Granda, Ospedale Maggiore Policlinico, 20142 Milan, Italy

**Keywords:** Health care, Dentistry, Dental radiology, Orthodontics, Paediatric dentistry

## Abstract

The aim of this study is to compare cone-beam computed tomography (CBCT) and bi-dimensional reconstructed lateral cephalograms (RLCs) in assessing mandibular body length and growth and to evaluate how mandibular reshaping influences the error in measuring mandibular body growth in bi-dimensional radiographs. Twenty-five patients with two CBCT scans taken at a mean distance of 2.21 ± 0.5 years were selected. The following measurements were performed: right and left mandibular body length at each point in time, mandibular growth, inter-gonial distance and mandibular symphyseal angle. From each CBCT, an RLC was obtained, and mandibular body length and growth were measured. Data analysis revealed a statistically and clinically significant difference in CBCT and RLC regarding the mandibular length of each patient at each point in time. However, mandibular growth was almost identical. A linear regression was performed to predict growth distortion between RLCs and CBCT depending on the ratio between transverse and sagittal mandibular growth. The expected maximum and minimum distortion, however, appeared not to be significant. In fact, a second linear regression model and a Bland–Altman test revealed a strong correlation between measurements of average mandibular body growth by CBCT and RLCs. As the same distortion occurs in the first and second RLCs, bi-dimensional radiographs remain the method of choice in evaluating mandibular body growth.

## Introduction

Mandibular length is an important indicator of therapeutic success. In the literature, this variable has been measured using different radiographic techniques. In 1931, Broadbent^1^ described a method to study facial growth by lateral teleradiographs. Despite some drawbacks, such as variable magnification, different grades of distortion, and limited repeatability of head position, the technique of superimposing cephalometric tracings taken over time has been accepted for clinical and research purposes in orthodontics^2^. Several aspects of the growth of the maxillofacial complex have been studied so far, such as the direction and intensity of growth in different cohorts of patients with a sequence of cephalometric radiographs^2–4^.

The growth of the maxillofacial complex is steep during the first 4 years of life^5^, and it becomes flatter until puberty, when it becomes steeper again during the adolescent growth spurt^6^. The timing of maxillofacial growth differs between boys and girls; its onset and peak occur at ages 12 and 14 years, respectively, in boys and 9.5 and 11.5 years, respectively, in girls^6–8^.

Modifications that occur in the human mandible during growth were first studied by Bjork^9,10^ and Enlow^11,12^. Small pointed pins were implanted into the mandible and used as a fixed reference for evaluating bone growth. While useful baseline information was provided by this and subsequent studies^2,4^, the data were limited to two-dimensional (2D) measurements. One of the limitations of bi-dimensional measurement is difficulty in assessing true sagittal growth depending on the tri-dimensional modification of the facial skeleton between two points in time^13,14^.

Multi-slice computed tomography (MSCT) can precisely measure bony structures tri-dimensionally^14^. The radiation dose delivered to the patient is consistently high, however, and the use of this technique is therefore extremely limited^15,16^. The development of cone-beam CT (CBCT), a less expensive alternative to MSCT that offers the same accuracy in tri-dimensional measurements of the facial skeleton and a lower radiation dose, has allowed researchers to begin studying mandibular growth in animal models^14^. Kim et al.^17^ were the first to evaluate mandibular growth with this technology, using it on New Zealand white rabbits. Major concerns about their work exist in regard to the relatively large time intervals between CBCT examinations, simplification in describing growth with chosen variables and the differences in mandibular gross morphology between rabbits and humans^18^. The considerably high radiation dosage compared to conventional bi-dimensional radiographs still makes CBCT unsuitable for the study of human mandibular growth or for diagnostic purposes, except where a routine CT scan would otherwise be necessary (i.e., Apert syndrome)^19,20^.

**Figure 1:**
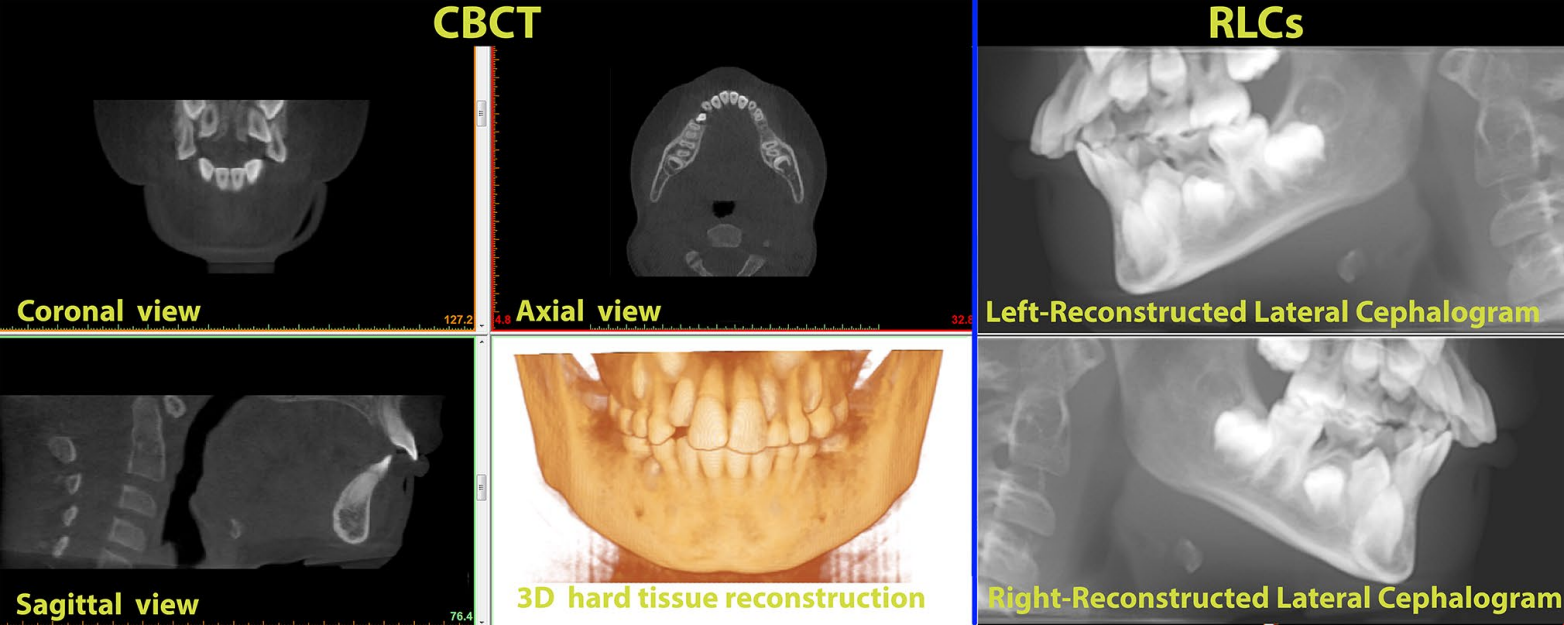
Example of the mandibular body of the same specimen for the two imaging methods. *CBCT* cone-beam computed tomography, *LC* lateral cephalogram.

Lateral radiographs are still the method of choice to understand normal growth and craniofacial development; to diagnose malocclusions and other facial abnormalities; and to quantify the effects of orthodontic, orthopaedic and surgical interventions^21,22^. However, latero-lateral teleradiographs are a two-dimensional (2D) image of a three-dimensional (3D) structure. Projection and superimposition of anatomical structures on a plane caused by an X-ray beam produce various grades of distortion, depending on several factors, such as the distance between each body part and the radiographic sensor, non-coplanarity between the radiographic sensor and each structure, and similar variables^14^. Moreover, the flattening and overlapping of bony structures makes it difficult to locate cephalometric points^23^. Lateral cephalograms can also be obtained from CBCT scans as reconstructed lateral cephalometric radiographs (RLCs, Fig. 1). The accuracy of linear and angular measurements evaluated with lateral cephalograms and RLCs showed no significant difference^24^.

### Specific objective

The aim of this study is twofold. First, we compared tri-dimensional CBCT and bi-dimensional RLCs in assessing mandibular body length and growth in skeletal class 2 subjects during pubertal growth spurt. Second, we evaluated how tri-dimensional mandibular reshaping during growth could affect distortion in the assessment of mandibular body growth with bi-dimensional radiographs.

## Result

Descriptive statistics and two-sample paired *t* tests for all linear (mm) and angular (°) measurements before (T0) and after growth (T1) are shown in Table 1.

**Table 1:** Comparisons of the mandibular body length (Go–Me), inter-gonial distance (GoR–GoL) and mandibular symphyseal angle (GoR–Mê–GoL) before and after growth.

	T0		T1		ΔT1–T0		*p* value
	Mean	SD	Mean	SD	Mean	SD	
3D Go–Me R (mm)^a^	70.123	5.296	74.322	5.564	4.218	1.875	< 0.001
3D Go–Me L (mm)^a^	70.182	5.044	74.537	5.298	4.353	1.656	< 0.001
2D Go–Me R (mm)^a^	59.214	5.425	63.403	4.515	4.192	1.816	< 0.001
2D Go–Me L (mm)^a^	59.645	5.223	63.817	5.914	4.179	1.943	< 0.001
3D GoR–GoL (mm)^a^	77.851	5.694	81.326	5.982	3.476	1.729	< 0.001
3D GoR–Mê–GoL (°)^a^	66.303	4.649	65.445	5.398	− 0.865	2.414	0.103

A *p* value < 0.05 was considered statistically significant.

^a^Numerical data are given as the means and standard deviations of measurements of two time points and two investigators.

Descriptive statistics and two-sample paired *t* tests for all linear (mm) and angular (°) measurements between RLCs and CBCT are shown in Table 2.

**Table 2:** Comparisons of the mandibular body length (Go–Me) and changes (ΔGo–Me) after growth between the 2 imaging methods.

	Baseline							After growth							Growth (changes)				
	3D CBCT (N = 25)		2D RLC (N = 25)		ΔCBCT-RLC		*p* value	3D CBCT (N = 25)		2D RLC (N = 25)		ΔCBCT-RLCs		*p* value	3D CBCT		2D RLC		*p* value
	Mean	SD	Mean	SD	Mean	SD		Mean	SD	Mean	SD	Mean	SD		Mean	SD	Mean	SD	
Go–Me R (mm)^A^	70.123	5.296	59.214	5.425	11.914	1.743	< 0.001	74.322	5.564	63.403	4.515	12.124	2.141	< 0.001	4.218	1.875	4.192	1.816	0.824
Go–Me L (mm)^A^	70.182	5.044	59.645	5.223	11.547	1.856	< 0.001	74.537	5.298	63.817	5.914	11.707	2.178	< 0.001	4.353	1.656	4.179	1.943	0.247

A *p* value < 0.05 was considered as statistically significant.

^A^Numerical data are given as the means and standard deviations of measurements of two time points and two investigators.

A statistically significant difference was found between mandibular body length assessed at T0 and at T1 (Table 1) in each group (RLCs and CBCT) and between bi-dimensional and tri-dimensional measurements at each point in time (*P* < 0.001) (Table 2). Mandibular growth (ΔGo–Me) as measured with the two imaging methods (RLCs and CBCT) was compared, and no statistically significant difference was found between the results (Table 2). The mandibular symphyseal angle (GoR–Mê–GoL) showed no statistically significant difference during growth. The inter-gonial distance (GoR–GoL) showed a statistically significant difference during growth (Table 2).

Measurement precision, assessed using a two-sample test for the coefficient of variation, showed no statistically significant difference between CBCT and RLCs (Table 3).

**Table 3:** Coefficient of variation and intra-observer and inter-observer agreement for: mandibular body length (Go–Me) and mandibular body growth (ΔGo–Me) for both imaging methods; Mandibular symphyseal angle (GoR–Mê–GoL) and inter-gonial distance (GoR–GoL) for CBCT.

	Coeff var				ICC	
	Mean ± SD	95% IC lower	95% IC upper	*P* value^b^	Intra-observer (Obs. I; Obs. II)	Interobserver^a^
**Go–Me R T1**						
CBCT	0.098 ± 0.005	0.085	0.111	0.314	0.995; 0.989	0.984
RLC	0.120 ± 0.011	0.093	0.148		0.988; 0.982	0.963
**Go–Me R T2**						
CBCT	0.094 ± 0.007	0.076	0.111	0.427	0.995; 0.997	0.981
RLC	0.118 ± 0.017	0.076	0.160		0.995; 0.989	0.956
**Go–Me L T1**						
CBCT	0.091 ± 0.006	0.077	0.104	0.201	0.994; 0.991	0.986
RLC	0.116 ± 0.011	0.089	0.142		0.988; 0.975	0.967
**Go–MeL T2**						
CBCT	0.089 ± 0.006	0.073	0.105	0.428	0.992; 0.996	0.982
RLC	0.121 ± 0.014	0.087	0.156		0.991; 0.984	0.964
**Δ Go–Me L growth**						
CBCT	0.428 ± 0.011	0.362	0.414	0.593	0.966; 0.972	0.918
RLC	0.524 ± 0.089	0.303	0.745		0.951; 0.924	0.875
**Δ Go–Me R growth**						
CBCT	0.488 ± 0.044	0.378	0.598	0.965	0.955; 0.965	0.957
RLC	0.451 ± 0.046	0.337	0.566		0.956; 0.938	0.887
**GoR–Mê–GoL T1**						
CBCT	0.092 ± 0.005	0.079	0.106		0.966; 0.953	0.984
**GoR–Mê–GoL T2**						
CBCT	0.101 ± 0.007	0.084	0.118		0.992; 0.974	0.988
**GoR–GoL T1**						
CBCT	0.096 ± 0.004	0.081	0.109		0.952; 0.967	0.981
**GoR–GoL T2**						
CBCT	0.099 ± 0.006	0.084	0.111		0.987; 0.947	0.979

*CBCT* cone-beam computed tomography, *RLC* reconstructed lateral cephalogram, *Coeff Var* coefficient of variation, *ICC* intraclass correlation coefficient, *Obs* observer, *MBL* mandibular body length.

^a^ nter-observer ICC data are given as the means of measurements at two time points and by two investigators.

^b^Two-sample test for coefficient of variation.

Each observer showed very high intra-observer agreement for CBCT measurements, with average (± standard deviation (SD), range) intra-observer intra-class correlation coefficients (ICCs) of 0.982 (± 0.016, 0.955–0.995) for observer I and 0.979 (± 0.016, 0.953–0.997) for observer II. Similar intra-observer ICCs were observed for the RLC counterparts, with mean values (± SD, range) of 0.978 (± 0.017, 0.951–0.995) for observer I and 0.964 (± 0.023, 0.924–0.989) for observer II.

Inter-observer agreement was excellent for CBCT with an average (± SD, range) ICC of 0.972 (± 0.024, 0.988–0.918). In comparison, inter-observer agreement for RLC was also very high but slightly lower than that of CBCT, with an average (± SD, range) ICC of 0.935 (± 0.04, 0.875–0.967). The intra-observer and inter-observer ICCs for all measurements are shown in Table 3.

Mean differences, range of 95% limits of agreement and 95% limits of agreement measured on CBCT and RLCs were calculated for mandibular length (right and left) and for mandibular growth (right and left). The results showed a low level of agreement in the linear measurement of mandibular body length at each point in time, whereas a high level of agreement was attested in mandibular body growth (ΔGo–Me). The results are reported in Table 4.

**Table 4:** Bland–Altman values on the differences between the measurements on CBCT and LCR.

	Go–Me right T0	Go–Me left T0	Go–Me right T1	Go–Me left T1	ΔGo–Me left	ΔGo–Me right	ΔGo–Me average
Mean difference	11.546	11.703	11.913	11.899	− 0.374	0.026	− 0.173
Range 95% limits of agreement	7.268	8.538	6.833	7.251	1.874	2.338	1.252
95% limits of agreement	15.173–7.905	15.977–7.434	15.336–8.502	15.515–8.267	− 1.304 to 0.576	1.192 to − 1.144	0.431 to − 0.772

Mean differences (mm), range of 95% limits of agreement (mm), and 95% limits of agreement (mm) for mandibular body length (Go–Me) and mandibular growth (ΔGo–Me).

**Figure 2:**
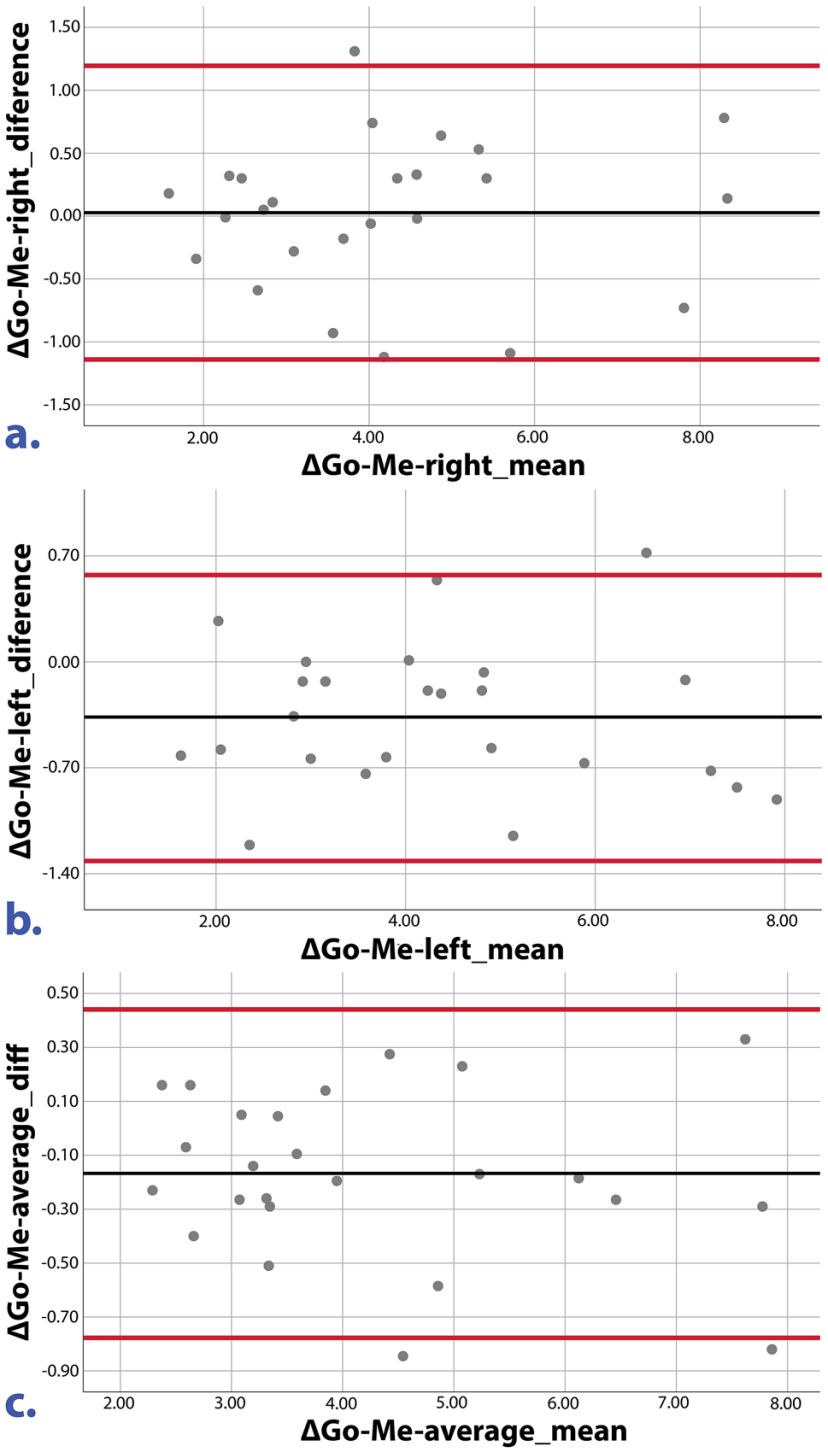
Bland–Altman plots for mandibular body growth comparison between 3D CBCT and 2D RLC measurements. Example Bland–Altman plots for mandibular body growth values of the right (**a**) and left (**b**) sides and the average (**c**) are shown. Black lines represent the mean of all differences (bias), and red lines represent the 95% limits of agreement between the 3D values and the measurements on 2D RLC.

Bland–Altman plots of right, left and average mandibular growth are reported in Fig. 2a–c.

One simple linear regression was calculated to predict Δ_2D–Go–Me based on Δ_3D–Go–Me.

**Figure 3:**
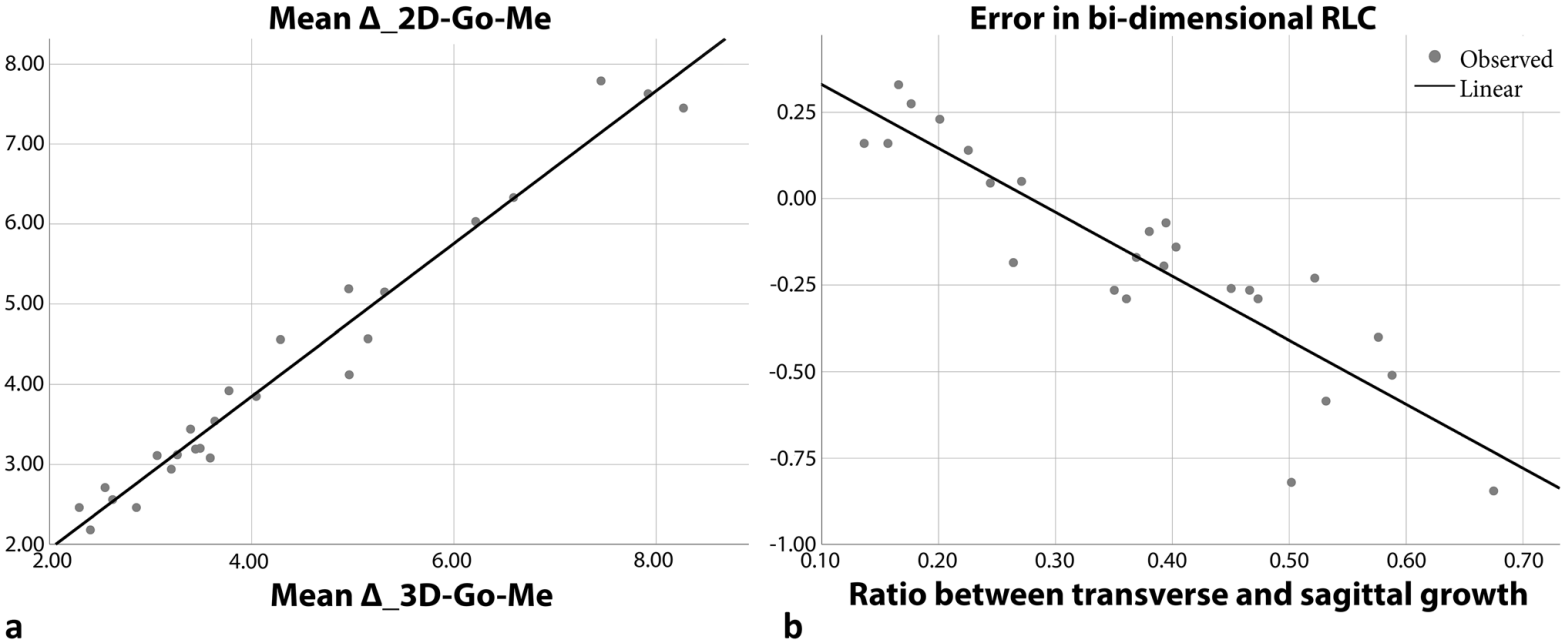
**(a)** Linear regression model showing the high concordance between 3D CBCT and 2D RLC mandibular body growth measurements. (**b**) Linear regression model highlighting relationship between the distortion of bi-dimensional radiographs RLCs compared with CBCT scan in the evaluation of mandibular growth (dependent variable, ΔGo–Me 3D − ΔGo–Me2D) and mandibular growth pattern based on the relationship between transverse and sagittal growth of the mandible (independent variable, ΔBO/ΔAO).

A significant regression equation was found (F(1,23) = 710.03, *p* < 0.001), with R^2^ = 0.969. The predicted value of Δ_2D–Go–Me is equal to 0.032 mm + 0.954 * (Δ_3D–Go–Me) mm (Table 5, Fig. 3a).

**Table 5:** Linear regression analysis: 1. Correlation between measurements of average mandibular body growth of each patient on CBCT (independent variable, Δ_3D Go–ME) and RLCs (dependent variable, Δ_2D Go–Me). 2. Correlation between transverse and sagittal growth of the mandible (independent variable, ratio of ΔBO to ΔAO) and error of bi-dimensional radiographs RLCs in the evaluation of mandibular growth compared with CBCT scan (dependent variable, ΔGo–Me3D – ΔGo–Me2D).

	Independent variable	Dependent variable	R	R square	*p* value		Unstandardized coefficients		Standardized coefficients	t score	Sign
							β	SE			
1	Δ_3D Go–Me	Δ 2D Go–Me	0.985	0.969	< 0.001	(Constant)	0.032	0.168	–	0.194	0.848
						Δ_3D	0.954	0.036	0.984	26.623	< 0.001
2	Ratio	Error	0.898	0.806	< 0.001	(Constant)	0.517	0.076	–	6.839	< 0.001
						Ratio	− 1.850	0.189	− 0.898	− 9.764	< 0.001

A second simple linear regression was calculated to predict the error in bi-dimensional RLC based on the ratio between transverse and sagittal growth (ΔBO/ΔAO).

A significant regression equation was found (F(1,23) = 95,341, *p* < 0.001) with R^2^ = 0.806. The predicted value of the error is equal to 0.517 mm + (− 1.850) + (ΔBO/ΔAO) mm (Table 5 and Fig. 3b).

## Discussion

The analysis of mandibular growth changes during the pubertal growth spurt has important consequences for the diagnosis and treatment planning of skeletal malocclusions. The majority of knowledge regarding facial growth comes from bi-dimensional cephalometric tracings and their superimposition^25–27^. Evaluation of growth and the changes caused by growth using bi-dimensional radiographs raise an important issue as a 3D object is flattened to a bi-dimensional image^14^.

The first aim of this study was to test whether mandibular growth in skeletal class 2 patients assessed on CBCT and on traditional 2D cephalometry showed any difference. The second was to test whether the error in bi-dimensional cephalograms was linked to the ratio between transverse and sagittal growth of the mandible. The results of the present study regarding average mandibular growth (Table 1) are in concordance with those published by Gomes and Lima^28^ that reported an annual mandibular body growth rate of as 2.16 mm/year during the pubertal growth spurt of skeletal class 2 patients.

The decision to select a sample of skeletal class 2 patients is because it appears to be the most frequently occurring malocclusion, especially in North America and Europe^29–31^. During orthodontic treatment, clinicians need to monitor mandibular growth, and this is enabled by longitudinal evaluation of lateral teleradiographs.

However, bi-dimensional measurements of craniofacial structures that do not lie on the mid-sagittal plane present various degrees of distortion, and the evaluation of growth could be affected. As suggested by Farronato et al.^32^ the comparison of the tracings from bi-dimensional radiographs and 3D CBCT scans shows that the measurement of the segment Go–Me (representing mandibular body) presents a statistically significant difference. Pittayapat et al.^33^ reported that cephalometric values measured on bi-dimensional radiographs are expected to be significantly different compared to those measured on CBCT. The segment Go–Me, representing mandibular body length, is the most affected by projection distortion^33^. This result can be explained by the fact that 2D radiographs are obtained by flattening a 3D object, thus projecting and distorting each point in proportion to its distance from the mid-sagittal plane. The mandibular body lies angled to the mid-sagittal plane, thus resulting in strong underestimation when projected on a bi-dimensional plane.

In this study, at each point in time, linear measurements of mandibular length were found to be significantly lower in RLCs than in CBCT by approximately 12 mm on both sides (Table 2).

To better understand the reason behind our choice to investigate this specific topic, one must consider the physiological pattern of mandibular growth. Mandibular growth has been extensively studied by researchers such as Enlow^11,12^, Franchi^26,34^ and Bjiork^3,9,10^. Significant morphological changes have been reported in the mandible during the peak of growth^35^. The available literature on the subject suggests that the mandible has different growth sites during growth spurt^3,10–12,26^. The main growth site is at the level of the mandibular condyle^3,10–12,26^.

The surface of the condyle grows with endochondral ossification, resulting in an increase mainly in the vertical dimension. Nonetheless, other sites are active during this period. For example, bone remodelling at the level of the mandibular body takes place at the level of the gonial angle; more precisely, the translation of the mandible forward and the increase in the sagittal dimension are due to the apposition of bone on the posterior surface of the mandibular ramus^3,9–12^. The mandibular body lengthens through the process of periosteal ossification with bone resorption on the anterior side of the mandibular ramus and contemporary bone apposition on the posterior surface of the mandibular ramus^3,9–12^. During the first years of life, the anterior surface of the ramus is located around the point where the first milk molar erupts^5,36^. The progressive bone resorption and apposition at the level of the ramus determines the growth of the body and the space available to accommodate the permanent molars. In the case of posterior dentoalveolar discrepancy and retention of mandibular molars, one possible explanation is indeed a reduced resorption at the level of the ramus^37^. As the growth of the mandibular body occurs posteriorly with divergent progression, it also involves an increase in the transverse dimension.

As mandibular growth appears to be a tri-dimensional phenomenon, the most accurate examination to assess mandibular body changes during adolescence is superimposing tri-dimensional volumes of the mandible obtained with a CBCT scan by tri-dimensional image registration based on the correspondence of voxels^34,38^. In fact, several authors are trying to identify stable points at the mandibular level to overlap the jaws and evaluate growth in a reliable way^34,39^. However, the justification and optimization principles that rule radiation exposure strongly limit the use of CBCT and therefore the available data as reported by the SEDENTEX and DIMITRA guidelines^16,40,41^. The potential benefits of this type of examination should be weighed against the increased exposure to ionizing radiation compared to conventional bi-dimensional imaging. Avoidance of unnecessary radiation exposure is crucial, particularly for children and adolescents^40,41^.

Contrary to the authors’ expectations, mandibular growth did not show any significant difference between RLCs and CBCT as shown in Table 2. This event seems to be attributable to the pattern of mandible growth. As reported in Table 1, the transverse growth of the mandible (ΔGoR–GoL) is similar to sagittal growth (ΔGo–Me), as indicated by the lack of a statistically significant difference between the angles GoR–Mê–GoL in the two points in time (see Table 1), thus showing a similar distortion in both RCLs. To further investigate the concordance of the measurements of mandibular growth in the two scans, a correlation analysis was carried out (see Table 5). Linear regression between mandibular growth in 3D and 2D (the independent and dependent variables, respectively) showed a very high correlation (R^2^ = 0.969), a beta coefficient close to 1 (Δ_3D coefficient = 0.984; *p* < 0.001) and a non-statistically significant constant (constant = 0.032 mm; *p* = 0.848). These values testify to how accurately RLCs are compared to CBCT and how little the values differ.

When growth is evaluated longitudinally, the least noticeable difference defines the threshold for growth detection. Our results show that measurements of the mandibular body length and its growth with CBCT are comparable to those of RLCs in terms of precision (see Table 3). Cephalometric measurements taken on RLCs were highly reliable, as inter-observer and intra-observer agreement was excellent and slightly lower than those measured on CBCT.

The Bland–Altman test for mandibular length showed that 95% limits of agreement of both imaging methods (CBCT, RLC) were from 15.17 to 15.97 mm for the upper bound and from 7.43 to 8.50 mm for the lower bound. Furthermore, mandibular body lengths assessed on CBCT were systemically larger than those measured on RLC. The average mean differences between CBCT and RLCs at T0 were 11.54 mm (right side) and 11.70 mm (left side); at T1, the average mean differences were 11.91 mm (right side) and 11.89 mm (left side). This difference is due to the bi-dimensional flattening of the skull in RLCs and its aforementioned consequences.

The Bland–Altman test for mandibular growth (Fig. 2 and Table 4) showed that the 95% limits of agreement of both imaging methods (CBCT, RLC) were instead − 1.14 mm (right side), − 1.31 mm (left side) and − 0.78 mm (on average) for the lower bound and 1.19 mm (right side), 0.57 mm (left side) and − 0.448 mm (on average) for the upper bound. The measurements of mandibular growth made with CBCT and RLC were very close: the mean differences were 0.03 (right side), − 0.37 mm (left side) and − 0.17 mm (average).

Since the measurements were performed by two operators, the evaluation of all four measurements (two measurements from each observer) in the Bland–Altman plot analysis considered the entire variation, thus making the limits of agreement wider and more realistic.

Considering the overall high concordance with CBCT and the low radiation exposure, lateral cephalometric analysis for the assessment and monitoring of mandibular growth can be performed by lateral cephalograms without any difference from the radio-diagnostic point of view.

Some authors are attempting to validate an MRI-based cephalometric protocol, which could have an important effect on treatment planning and the possibility of monitoring mandibular growth longitudinally, especially in the case of young patients, since MRI can be repeated due to the absence of ionizing radiation^42–45^. The current development and spread of radiation-free technologies in dentistry, such as MRI, will allow researchers to better understand maxillo-facial growth and physiology^46–48^.

**Figure 4:**
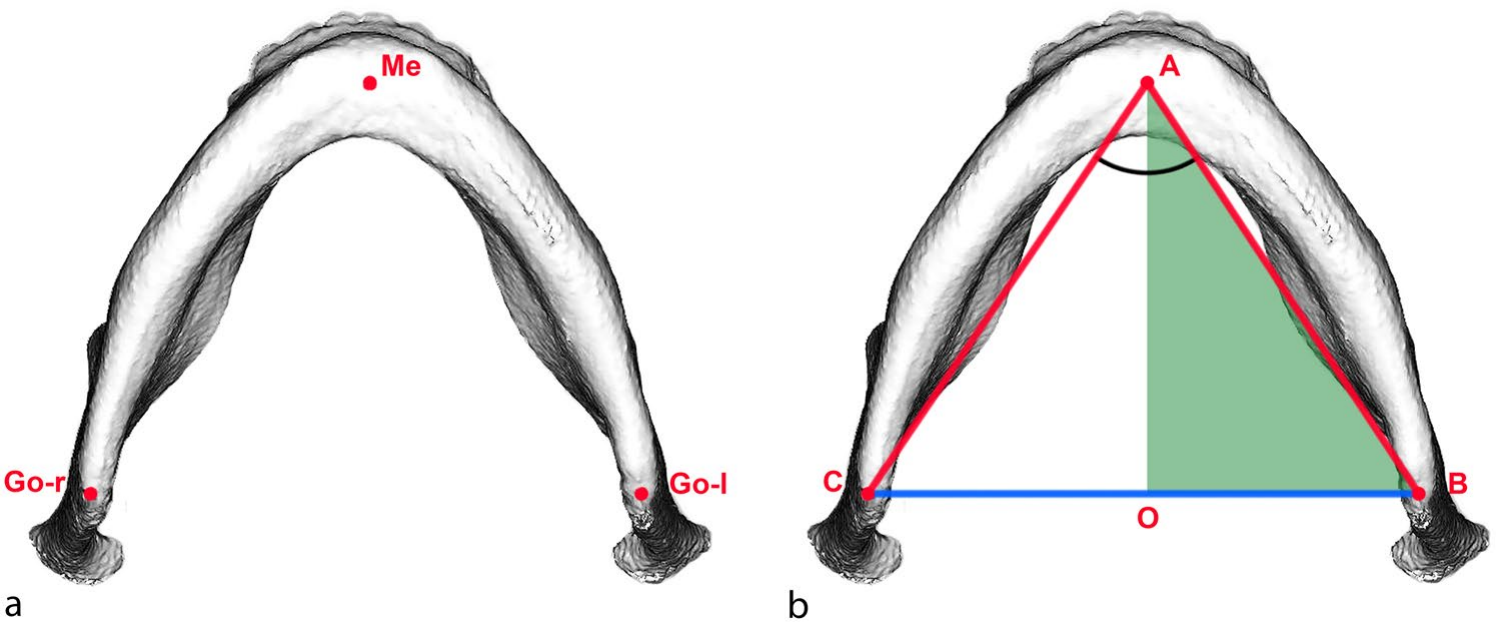
(**a)** Cephalometric landmarks used in the present study. For each CBCT and RLC dataset, 3 cephalometric landmarks were determined on multiplanar images. Definitions of cephalometric landmarks: GoL/GoR = left/right gonion (midpoint on the curvature of the angle of the mandible); Me = menton (most inferior point of mandibular symphysis). Definitions of cephalometric measurements: GoL/GoR-Me = mandibular body length right/left; GoL-GoR = inter-gonial distance; GoR–Mê–GoL = mandibular symphyseal angle. (**b**) Isosceles triangle (CAB) representing the mandible of the patient. The authors considered an isosceles triangle for each patient having the symmetrical sides (AB; AC). The isosceles triangles were divided in two equal right triangles (ABO). Definitions of the measurements: AB/AC (hypotenuse) = right/left mandibular body length; BC = inter-gonial distance; BO (cathetus) = half inter-gonial distance and transverse component of the mandible; AO (cathetus) = sagittal component of the mandible; CÂB = mandibular symphyseal angle; OÂB = half mandibular symphyseal angle.

Focusing on the other aim of this study, which is whether mandibular reshaping during pubertal growth spurt could interfere with mandibular growth assessment through bi-dimensional radiographs, the second linear regression has a twofold meaning. There is a meaningful relationship (R^2^ = 0.81) between the bi-dimensional error in assessing growth and the mandibular growth pattern (the ratio or transverse to sagittal growth). If the transverse component of growth is greater than the sagittal component, the resulting distortion in the second radiograph will lead to an underestimation of the real growth, as the projection of the mandibular body is affected by a greater OAB angle (Fig. 4). In contrast, if the reverse is true and the sagittal component is greater, the bi-dimensional growth will appear larger than the real growth assessed on CBCT. However, the OAB angle, which is half of the mandibular symphyseal angle (assuming these patients’ mandibles are almost perfectly symmetrical), does not show any statistically significant difference between the two points in time even though it appears that in the majority of patients, it trends toward a slight and not clinically significant reduction (− 0.86 ± 2.41). In fact, the second linear regression (coefficient of ratio = − 1.850, *p* < 0.001; constant = 0.517, *p* < 0.001) shows that in the cohort of patients who was analysed, the expected value of distortion in the assessment of mandibular growth ranges between − 0.732 and 0.265 mm, thus showing a result that is not clinically significant.

Lin et al.^49^ affirmed that significant errors exist in the measurements of mandibular body growth using 2D RLCs compared to CBCT in six miniature pigs. Clinical and statistically significant underestimation of the actual growth was reported. Lin et al.^49^ concluded that this event should be considered in orthodontic treatment planning to more precisely control the treatment process and outcome. However, the present study reveals that within its limitations, the actual linear mandibular body growth in humans during growth spurt is equally evaluated in entity and accuracy with bi-dimensional radiographs. These phenomena occur because humans and pigs have different mandibular development, and no generalization should be made based on their data. The development of the human mandible appears to be completely different, as it develops almost by the same amount in the sagittal and transverse planes, keeping the distortion between 2 and 3D stable at different points in time. It is therefore obvious that although the second regression analysis points to a close relation between growth pattern and projection distortion, the amount of error is of no relevance from any point of view. Bi-dimensional assessment of linear mandibular growth should therefore be performed accurately on lateral cephalograms, which are the absolute gold standard and have been for almost a century.

Conventional 2D imaging methods are inherently limited by anatomical superimpositions and inherent image distortion^14^. However, the authors confidently affirm, within the limits of the current study, that mandibular growth shows no clinical or statistical difference whether it is assessed on conventional lateral cephalograms or on CBCT.

Measured values appear equally reliable and precise. Although a difference exists, the difference in mandibular growth as assessed with the two imaging methods is of no clinical or statistical significance. The development of the human mandibular body through puberty appears to be almost equal in the transverse and sagittal planes, thus keeping distortion of the mandibular body similar between CBCT and conventional bi-dimensional radiographs at the two analysed points of time.

One of the weaknesses of the study is its sample size, which is small, albeit adequate to provide a sufficient level of confidence in the results in accordance to the sample size calculation (see dedicate section: “Statistical analysis and sample size calculation”). The present study investigates only the changes occurring during the peak of growth; further investigations should focus on morphological changes occurring before and after it. Another possible development of the present study could include the analysis of mandibular growth represented as the distance between the Condylion point (representing the highest and most posterior point on the contour of the mandibular condyle) and menton. However, the study design would be much more complex and could be affected by many biases, as it would be difficult to construct a simplified model that considers the influence of both the change in the angle of the menton and the change in the angle between the mandibular body and mandibular ramus. The analysis of the effect of facial orthopaedic treatment of mandibular shape could also prove to be of some interest. The evaluation of patients with different skeletal classes and those presenting various degrees of asymmetry would be beneficial. Such data would allow us to draw conclusions that could more easily be extended to the entire population, as the findings of the present study fit only people who present a satisfactory degree of facial symmetry.

## Methods

### Study design

A retrospective study was performed analysing mandibular CBCT scans of patients examined at the Department of Dentistry and Maxillofacial Surgery of Fondazione IRCCS Cà Granda Ospedale Maggiore Policlinico, Milano, Italy.

This study was approved by the appropriate Institutional Review Board (IRB) within the research project of the year 2018 O.U.N. 420/425 of Fondazione IRCCS Cà Granda Ospedale Maggiore Policlinico, Milano. Parents of each subject signed a proper informed consent form to allow the use of the records in anonymous form for research purposes. All procedures performed in this retrospective study involving human participants were in accordance with the ethical standards of the institutional committee and with the 1964 Helsinki declaration and its later amendments or comparable ethical standards. All methods were performed in accordance with relevant guidelines and regulations.

### Types of Participants and Inclusion Criteria

All patients were treated at the Department of Biomedical Surgical and Dental Sciences at the University of Milan, Fondazione IRCCS Cà Granda, Ospedale Maggiore Policlinico Milan, between January 2011 and December 2018. CBCT scans for this study were derived from previous research and clinical databases and were taken because of different conditions.

CBCT scans including at least all of the mandibular body were selected from the records archived at the Department of Dentistry of the same hospital.

Patients were selected on the basis of the following inclusion criteria:

1. Sufficient field of view (FOV) to include Gonion (R/L) and Menton;
2. Patients without congenital or acquired dentofacial deformities (for example cleft lip and palate, Apert syndrome, and post-traumatic deformity);
3. Patients without clinically significant asymmetry (2 mm difference between right and left mandibular body length);
4. Patients without history of oral and maxillofacial surgery;
5. Patients who had undergone 2 CBCTs at least 18 months apart;
6. Skeletal class 2 according to Steiner with ANB greater than 4°;
7. Patients who did not undergo any facial orthopaedic treatment;
8. Patients who had the two CBCTs including a relevant part of the pubertal growth spurt assessed with chronological age using as reference the limits reported by Mellion et al.^6^ as onset and peak for male (12–14 years old) and female subjects (9.5–11.5 years old). A range of 6 months, plus or minus each age limit, was considered acceptable.

A total of 1,500 medical records were evaluated. Twenty-five patients met the eligibility criteria with a mean distance between CBCT scans of 2.21 ± 0.5 years. The sample was composed of 12 males (mean age at first scan of 12, 3 ± 0.4 years and 14.2 ± 0.5 years at the second scan) and 13 females (mean age at first scan of 8.9 ± 0.3 years and 11.1 ± 0.5 years at the second scan).

### CBCT examination and postprocessing

For each patient in the study, a 3D volumetric image was obtained using the iCAT FLX V-17 Series cone-beam dental-imaging system (1910 N. Penn Road, Hatfield, PA 19440). The scanning protocol involved a voxel size of 0.4, a slice thickness of 0.4 mm, and an FOV from 9 × 11 cm to 16 × 8 cm, depending on the diagnostic question, to minimize radiation exposure. The scan protocol had a standard resolution of 360 rotations, 300 image frames, tube voltage of 120 kV [p], 5 mA, and scanning time of 3.7 s.

Patients’ heads were positioned in the iCAT FLX V-17 CBCT scanner (Imaging Sciences International, Inc, Hatfield, PA), with the Frankfurt horizontal plane parallel to the floor, using the midline light beam to coincide with the mid-sagittal plane passing through the glabella (natural head position).

The raw data from the CBCT scan were exported and reconstructed, coded, and converted into Digital Imaging and Communications in Medicine (Dicom3) file format. The Dicom3 files were then imported into Mimics Research version 20.0 to evaluate 3D mandibular body length (NV, Technologielaan 15, 3001 Leuven, Belgium, https://www.materialise.com/en/medical/mimics-innovation-suite/mimics) (Fig. 1). Reconstructed lateral cephalograms (RLCs) were obtained from CBCT by lateral radiographic projection of the entire volume using iCAT Vision software (Imaging Sciences International, Inc., https://ct-dent.co.uk/i-cat-vision/) (Fig. 1). Two RLCs (right and left projections) were reconstructed from each CBCT. All RLCs were imported into cephalometric analysis software (Dolphin Imaging Cephalometric and Tracing Software, version 11.9, Chatsworth, California, https://www.dolphinimaging.com/Media/DolphinNews?Subcategory_OS_Safe_Name=20160913).

### Measurements

Cephalometric analysis of 3D CBCT imaging and RLCs was performed.

For every CBCT scan, three points (Go right, Go left, and Me) were identified in Mimics. Three linear measurements and one angle were evaluated: right and left mandibular body length (GoR–Me, GoL–Me), inter-gonial distance (GoR–GoL) and mandibular symphyseal angle (GoR–Mê–GoL) (Fig. 4a).

The points (point Go right, Go left, Me) were marked using the 2D multiplanar reconstruction (MPR) images (axial, sagittal, and coronal slices). It is important that the points satisfy all of the description requirements in all three planes of space at the same time.

Three-dimensional mandibular body length is defined as the distance between the menton (Me), the most inferior midpoint of chin on the outline of the mandibular symphysis, and gonion (Go), the point of maximum convexity of the mandibular angle. The inter-gonial distance is defined as the distance between the right and left gonia. The mandibular symphyseal angle (GoR–Mê–GoL) was drawn between the left and the right gonion and menton (Fig. 4a).

Bi-dimensional mandibular body length was assessed on RLCs tracings using corresponding bi-dimensional points (Go and Me) with cephalometric analysis software (Dolphin Imaging cephalometric and tracing software, Chatsworth, California). Am obvious limitation was that neither the mandibular symphyseal angle nor the inter-gonial distance was measured because they cannot be evaluated on RLCs.

Mandibular body growth was determined as the difference between mandibular body length at the two points in time. Mandibular symphyseal angle variation was assessed as the difference between the angles measured at the two points in time.

All measurements were taken by two expert clinicians with a postgraduate specialization in orthodontics (CM, FB), and both experienced in tri-dimensional and bi-dimensional dental imaging for orthodontic purposes. After 4 weeks, the same assessments were repeated by both examiners to assess intra-operator and inter-operator reliability. Observers were blinded to patients’ identities.

### Study procedure

A simplified model was created to evaluate the error of bi-dimensional RLCs in assessing mandibular body growth (ΔGo–Me). As mentioned above, 2D cephalometric radiographs in diagnosis and treatment planning are limited due to geometric distortions occurring when a tri-dimensional structure is flattened on a bi-dimensional film. Mandibular body length undergoes a significant distortion, as the gonion points do not lie on the mid-sagittal plane^33^.

During mandibular growth, this distortion may be affected by the variation in mandibular symphyseal angle and inter-gonial distance, resulting in an overestimation or underestimation of the real mandibular body growth. If the menton and the two gonion points (left and right) are considered, the 3D mandible can be simplified as a triangle (Fig. 4b).

The rationale behind selecting symmetric patients is to obtain a simplified model of an isosceles triangle. Left and right 3D mandibular body lengths are not clinically different and can be averaged to their mean without a clinically relevant alteration of the real value. For each patient, the authors defined an isosceles triangle whose symmetrical sides (AB; AC) were equal to the mean of the left and right mandibular body 3D length, and the angle between them was equal to the symphyseal angle (GoR–Mê–GoL = CÂB). The third side of the triangle equalled the inter-gonial distance (BC) (Fig. 2b).

Each isosceles triangle was divided into two equal right triangles (Fig. 2b). Only one right triangle per patient was considered (ABO). Each right triangle was characterized by a hypotenuse that equalled the mean 3D distance for each patient between the gonion and menton (AB); an angle OÂB that equalled half the patient’s symphyseal angle GoR–Mê–GoL (CÂB/2); a cathetus that equalled the distance between the menton and the segment GoR–oL in the original isosceles triangle and represented the sagittal component of the mandible (AO); and another cathetus that represented the transverse component of the mandible and equalled half the inter-gonial distance (1/2 * GoR–GoL = BO) (Fig. 2b).

For each patient, a right triangle at T0 and a right triangle at T1 were constructed. The ratio between the difference between BO at T1 and BO at T0 and the difference between AO at T1 and AO at T0 (ΔBO/ΔAO) represented the ratio of the transverse and sagittal components of mandibular body growth.

The difference between 3D mandibular length and 2D mandibular length at T1 minus the difference of the same variables at T0 represented the quantity of overestimation or underestimation each patient presented in evaluating growth with 2D imaging (ΔGo–Me3D − ΔGo–Me2D).

### Statistical analysis and sample size calculation

A preliminary analysis was run by G*Power (version 3.1.9, https://www.psychologie.hhu.de/arbeitsgruppen/allgemeine-psychologie-und-arbeitspsychologie/gpower.html) on 10 subjects to obtain data for power analysis evaluation. The values of the mean difference in mandibular body growth between CBCT and RLCs were used to perform the power analysis calculation along with the corresponding SDs. The data used to perform the analysis were as follows: mean difference = 0.305; σ = 0.48. The results of the power analysis indicated that to reach 90% power, 23 subjects were needed.

Statistical analysis was performed with SPSS software (version 25.00; IBM Corp, Armonk, NY, https://www.ibm.com/support/pages/release-notes-ibm®-spss®-statistics-250).

The mean and SD were calculated for each variable. The computation of the means and SDs were based on the four linear measurement values (two measurements by each of the two observers). Shapiro–Wilk tests were used to check whether the data were normally distributed. In the case of non-normally distributed values, log-transformation was used to ensure a normal distribution.

- The difference between two points in time was assessed for bi-dimensional and three-dimensional mandibular body growth, inter-gonial distance and mandibular symphyseal angle.
- The difference between the two imaging methods in the assessment of mandibular body length at both time points and in mandibular body growth was analysed with a paired *t *test.
- To evaluate and compare the precision of the measurements, the coefficient of variation was separately computed for the GoR/L–Me and ΔGo–Me for the two methods and at two time points. The computation of the means and SD were based on the four linear measurement values (two measurements by each of the two observers).
- A two-sample test for the coefficient of variation was used to determine whether there was a significant difference between the coefficient of variation of the two imaging methods^50^.
- For all measurements, intra- and inter-observer reliability were assessed using an ICC.
- Bland–Altman analysis was used to evaluate the agreement between RLCs and CBCT values for each measurement with average values of the two time points and two investigators, with 95% limits of agreement. The ranges for the 95% limits of agreement were provided^51^.
- Two linear regression models were employed to assess the correlation between measurements of average mandibular body growth of each patient in CBCT (independent variable, ΔGo–Me) and RLCs (dependent variable, ΔGo–Me) and to predict distortion of bi-dimensional radiographs RLCs in the evaluation of mandibular growth compared with CBCT scan (dependent variable, ΔGo–Me 3D—ΔGo–Me2D) based on the relationship between transverse and sagittal growth of the mandible (independent variable, ΔBO/ΔAO).

Statistical test results with *P* values less than 0.05 were considered statistically significant.

## Data availability

The datasets analyzed during the current study are available from the corresponding author on reasonable request.
